# Quantification of disease progression in spinal muscular atrophy with muscle MRI—a pilot study

**DOI:** 10.1002/nbm.4473

**Published:** 2021-01-22

**Authors:** Louise A.M. Otto, Martijn Froeling, Ruben P.A. van Eijk, Fay‐Lynn Asselman, Renske Wadman, Inge Cuppen, Jeroen Hendrikse, W‐Ludo van der Pol

**Affiliations:** ^1^ Department of Neurology, UMC Utrecht Brain Center University Medical Center Utrecht, Utrecht University Utrecht The Netherlands; ^2^ Department of Radiology University Medical Center Utrecht, Utrecht University Utrecht The Netherlands; ^3^ Biostatistics & Research Support, Julius Center for Health Sciences and Primary Care University Medical Center Utrecht, Utrecht University Utrecht The Netherlands; ^4^ Department of Neurology and Child Neurology, UMC Utrecht Brain Center University Medical Center Utrecht, Utrecht University Utrecht The Netherlands

**Keywords:** MRI, skeletal muscle, spinal muscular atrophy

## Abstract

**Objectives:**

Quantitative MRI (qMRI) of muscles is a promising tool to measure disease progression or to assess therapeutic effects in neuromuscular diseases. Longitudinal imaging studies are needed to show sensitivity of qMRI in detecting disease progression in spinal muscular atrophy (SMA). In this pilot study we therefore studied one‐year changes in quantitative MR parameters in relation to clinical scores.

**Methods:**

We repeated quantitative 3 T MR analysis of thigh muscles and clinical testing one year after baseline in 10 treatment‐naïve patients with SMA, 5 with Type 2 (21.6 ± 7.0 years) and 5 with Type 3 (33.4 ± 11.9 years). MR protocol consisted of Dixon, *T*_2_ mapping and diffusion tensor imaging (DTI). The temporal relation of parameters was examined with a mixed model.

**Results:**

We detected a significant increase in fat fraction (baseline, 38.2% SE 0.6; follow‐up, 39.5% SE 0.6; +1.3%, *p* = 0.001) in all muscles. Muscles with moderate to high fat infiltration at baseline show a larger increase over time (+1.6%, *p* < 0.001). We did not find any changes in DTI parameters except for low fat‐infiltration muscles (m. adductor longus and m. biceps femoris (short head)). The *T*_2_ of muscles decreased from 28.2 ms to 28.0 ms (*p* = 0.07). Muscle strength and motor function scores were not significantly different between follow‐up and baseline.

**Conclusion:**

Longitudinal imaging data show slow disease progression in skeletal muscle of the thigh of (young‐) adult patients with SMA despite stable strength and motor function scores. Quantitative muscle imaging demonstrates potential as a biomarker for disease activity and monitoring of therapy response.

## Graphical abstract

Quantitative 3T MRI of thigh muscle in spinal muscular atrophy patients detected a significant increase in fat fraction and decrease in T2 over the course of one year. We did not find any changes in the DTI parameters MD and FA except for low fat‐infiltration muscles (m. adductor longus and m. biceps femoris (short head)). Muscle strength and motor function scores remained stable. Quantitative muscle MRI demonstrates potential as a biomarker for disease activity and monitoring of therapy response.

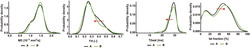

**Abbreviations**
DTI: diffusion tensor imaging
FA: fractional anisotropy
HFMSE: Hammersmith Functional Motor Scale, Expanded
HHD: hand‐held dynamometry
MD: mean diffusivity
MRC: Medical Research Council
PCA: principal component analysis
qMRI: quantitative MRI
SMA: spinal muscular atrophy
SNR: signal to noise ratio
SPAIR: spectral attenuated inversion recovery
SPIR: spectral presaturation with inversion recovery

## 1 INTRODUCTION

Hereditary proximal spinal muscular atrophy (SMA) is the leading genetic cause of death in infancy and severe impairment in childhood and later life. It is caused by loss of function of the survival motor neuron (*SMN*) 1 gene and characterized by abnormalities and dysfunction of motor neurons, neuromuscular junction and muscle tissue.^1^, ^2^, ^3^, ^4^, ^5^

The first genetic therapies for SMA were introduced in the past five years with prospects for additional therapies in the near future. Clinical trials have shown that genetic therapies improved infantile survival and motor function in both babies and young children.^6^, ^7^ Assessment of treatment efficacy in older children and adults is complicated by the relatively slow progression of the decline of muscle strength and motor function in patients with SMA.^8^, ^9^, ^10^, ^11^, ^12^ Sensitive biomarkers to detect decline caused by disease progression and early response to treatment are necessary to evaluate treatment effects at a relatively early stage of treatment to minimize risk and burden to patients and to optimize cost‐efficiency.

Quantitative MRI (qMRI) of muscles is a promising tool to measure disease progression or to assess therapeutic effects in neuromuscular diseases, including SMA.^13^, ^14^, ^15^, ^16^, ^17^, ^18^, ^19^, ^20^, ^21^, ^22^ We recently reported several unique qMRI characteristics in a cross‐sectional study on patients with SMA Types 2 and 3. First, quantification of fat infiltration in patients with SMA differentiates between vulnerable and resilient thigh muscles. Second, the bias of fat infiltration resulted in a slightly decreased *T*_2_. Finally, diffusion tensor imaging (DTI) showed decreased mean diffusivity (MD) in combination with increased fractional anisotropy (FA), which may reflect muscle atrophy.^23^ Longitudinal imaging studies are needed to show sensitivity in detecting disease progression. Especially with experimental methods such as qMRI there is a need for reference data, preferably of treatment‐naïve patients. As treatment is becoming available for patients, there is subsequent less opportunity to obtain data on the natural history of disease progression. We repeated qMRI analysis of thigh muscles in treatment‐naïve patients with SMA one year after baseline. We here report on the novel methodology required for analysis of longitudinal imaging data and present the course of qMRI and clinical measures over time. Our data show that qMRI can serve as a biomarker for disease progression.

## 2 METHODS

### 2.1 Study population

**FIGURE 1:**
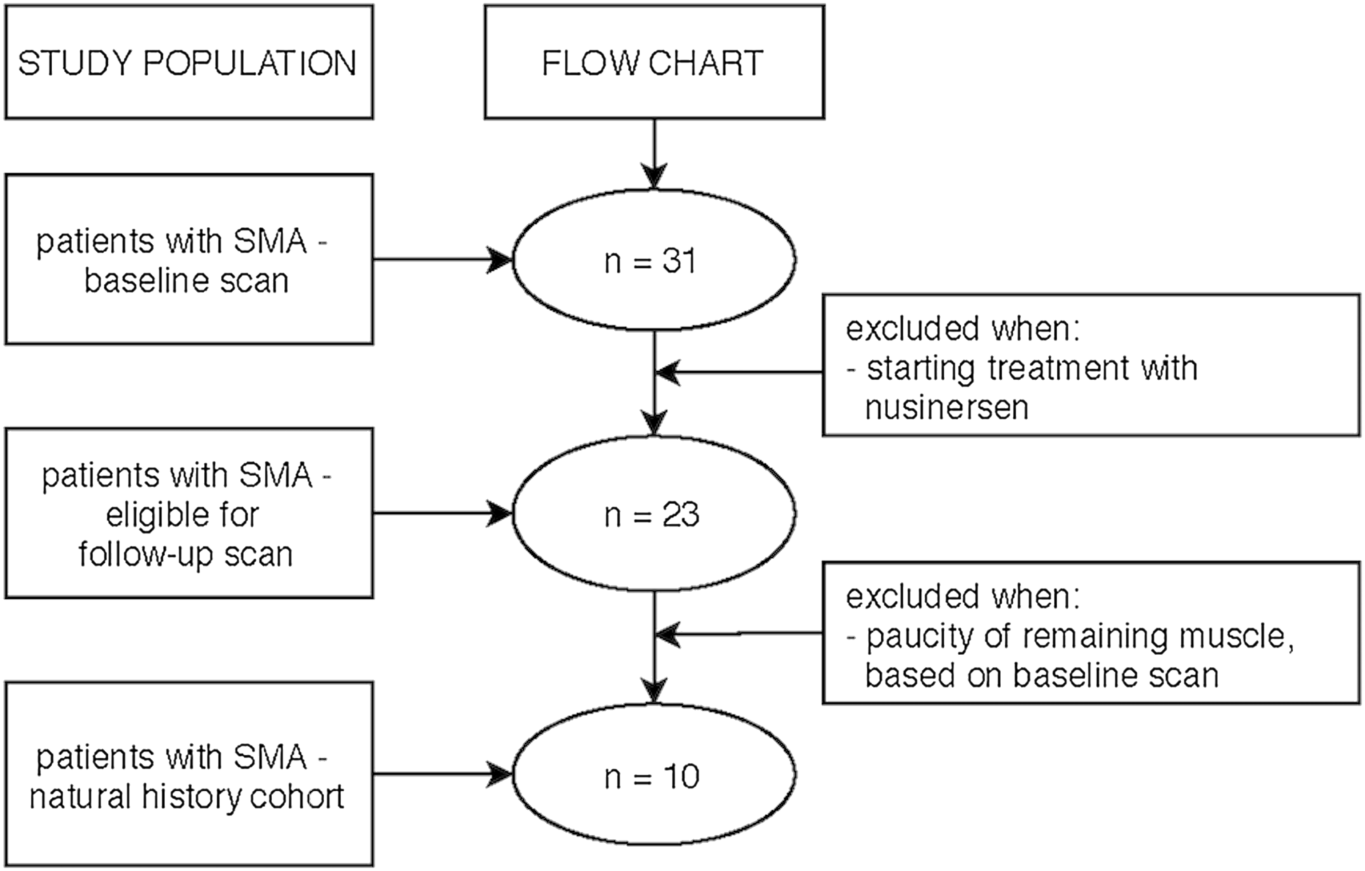
Flowchart of study inclusion and follow‐up procedure

We invited 31 patients who participated in our prospective baseline study for a follow‐up MRI after one year. Exclusion criteria were any type of invasive ventilation, a postural change of more than 15% in forced vital capacity between sitting and supine position, orthopnea, pronounced swallowing problems, pregnancy, non‐MR compatible material in the body or any contra‐indication for 3 T MR. Additional exclusion criteria for participation were severe fatty infiltration of muscle tissue after visual evaluation at baseline, the use of any of the available SMA therapies or participation in a clinical trial (Figure 1). Ten patients were eligible (three males and seven females). Five patients had SMA Type 2 (mean age 21.6 ± 7.0 years) and five SMA Type 3 (mean age 33.4 ± 11.9 years; see Table 1). The study was approved by the local ethics committee (no 17‐226/NL61066.041.17) and was conducted in accordance with the Declaration of Helsinki.

**TABLE 1:** Clinical characteristics

Clinical characteristics	SMA Type 2	SMA Type 3
*N*	5	5
Age in years: mean [range]	21.6 [15.7‐34.9]	33.4 [18.8‐52.8]
Sex (M:F)	0:5	3:2
*SMN2* copy number		
3	4	0
4	1	4
5	0	1
Disease duration in months: mean (SD)	250 (82)	234 (120)
Ambulatory status *n* (%)	0 (0)	5 (100)

F, female; M, male; *N*, number; SD, standard deviation; *SMN2*, survival motor neuron 2 gene.

Clinical characteristics are reported for patients with SMA Type 2 and Type 3.

### 2.2 Clinical evaluation

We assessed motor function with the Hammersmith Functional Motor Scale, Expanded (HFMSE) (range 0‐66; a lower score indicates poorer motor ability and function).^24^, ^25^ Muscle strength was documented with the Medical Research Council (MRC) scoring system (MRC, 1976).^26^ Additionally, we performed hand‐held dynamometry (HHD) of the adductors, quadriceps and hamstring muscles on both sides with a MicroFET2 device (Hoggan Health Industries, Salt Lake City, UT, USA). All clinical measurements were performed at baseline and follow‐up by the same trained evaluator (LAMO, two years of experience). The clinical evaluation followed directly after MR examination.

### 2.3 MR acquisition

All MR examinations were performed on the same 3 T MR scanner (Philips Ingenia, Philips Medical Systems, Eindhoven, The Netherlands) and according to the same protocol as performed at baseline with supine position and feet first, using a 12‐channel posterior and 16‐channel anterior body coil. The field of view was set according to the position of the image stack of the first scan, which was positioned approximately 175 mm below the femoral head.

**FIGURE 2:**
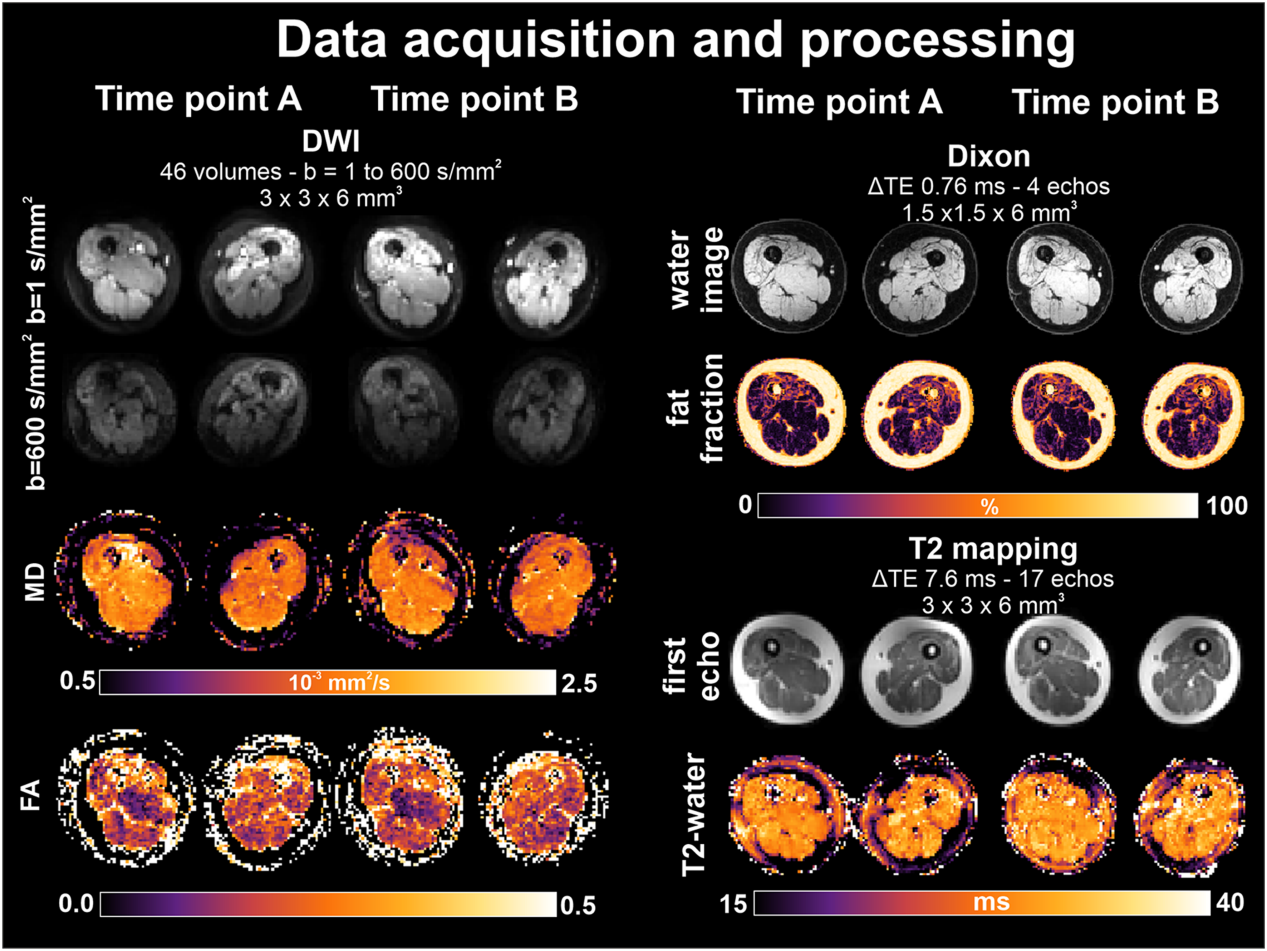
**Data acquisition and processing of qMRI parameters** Legend: diffusion weighted imaging; FA= fractional anisotropy; mm= millimeter; ms = millisecond; MD = mean diffusivity; TE = echo time; s= second. One subject is highlighted to visually present the dataset at time‐point A and time‐point B (alongside); for each of the parameters the raw data is projected above the processed data. The specifications of the 3 sequences are given

The MR protocol comprised a four‐point Dixon sequence (*T*_R_/*T*_E_/210/2.6/3.36/4.12/4.88 ms; flip angle 10°; voxel size 6 × 1.5 × 1.5 mm^3^; no gap; 25 slices); *T*_2_ mapping (17 echoes *T*_R_/*T*_E_/Δ*T*_E_ 4598/17/7.6 ms; flip angle 90/180°; voxel size 6 × 3 × 3 mm^3^; slice gap 6 mm; 13 slices, no fat suppression) and DTI spin echo echo‐planar imaging (*T*_R_/*T*_E_ 5000/57 ms; *b*‐values 0 (1), 1 (6), 10 (3), 25 (3), 100 (3), 200 (6), 400 (8) and 600 (12) s/mm^2^; voxel size 6 × 3 × 3 mm^3^; no gap; 25 slices, spectral attenuated inversion recovery (SPAIR) and spectral presaturation with inversion recovery (SPIR) fat suppression (Figure 2). The total scan time was about ~10 min. The MR protocol has been validated in a previous multicenter study for reproducibility and high temporal stability.^27^ See Table 2 for the acquisition parameters.

**TABLE 2:** Acquisition parameters of MR protocol. Specifications per sequence at a field strength of 3 T

	4‐point Dixon	*T*_2_ mapping	DWI
Sequence	multi‐acquisition gradient echo	multi‐echo spin echo	spin‐echo echo‐planar imaging
Repetition time (ms)	210	4598	5000
Echo time (ms)	2.6/3.36/4.12/4.88	17 × 7.6	57
Flip angle (°)	10	90/180	90/180
Acquisition matrix	320 × 320		160 × 92
FOV	480 × 480		480 × 276
Resolution (mm^2^)	1.5 × 1.5	3 × 3	3 × 3
Slices	25	13	25
Slice thickness (mm)	6	6	6
Slice gap (mm)	0	6	0
*b*‐values (no of images) (mm/s^2^)		0	0 (1), 1 (6), 10 (3), 25 (3), 100 (3), 200 (6), 400 (8), 600 (12)
Fat suppression			gradient inversion + SPAIR (main fat signal) + SPIR (olefinic fat signal)
SENSE/partial Fourier	2/1	2/1	1.9/0.75
Acquisition time (min:s)	1:20	3:05	3:30

DWI, diffusion‐weighted imaging. SENSE, sensitivity encoding.

### 2.4 MR processing

We processed MR data using the custom toolbox QMRITools for Mathematica (mfroeling.github.io/QMRITools). All data were checked visually for data quality and motion artifacts (MF and LAMO, 12 years and 2 years experience, respectively). The processing steps have been described previously.^23^, ^27^ In short, Dixon data were reconstructed using an IDEAL method with the estimation of *B*_0_ and *T*_2_*, *T*_2_ mapping was processed with extended phase graph fitting,^28^ and DTI with a fitting method (iterative weighted linear least squares (iWLLS) with robust extraction of kurtosis indices with linear estimation (REKINDLE) outlier detection), denoised with the principal component analysis (PCA) method and corrected for eddy current distortion and subject motion; the signal to noise ratio (SNR) of the DTI data was obtained using the PCA denoising algorithm (Figure 2).^29^

Manual segmentation of all datasets was done by one researcher (LAMO, two years of experience). All muscles were segmented for all slices of the imaging stack. However, not all muscles could be segmented when they were no longer clearly present.

### 2.5 Comparison of imaging stacks

**FIGURE 3:**
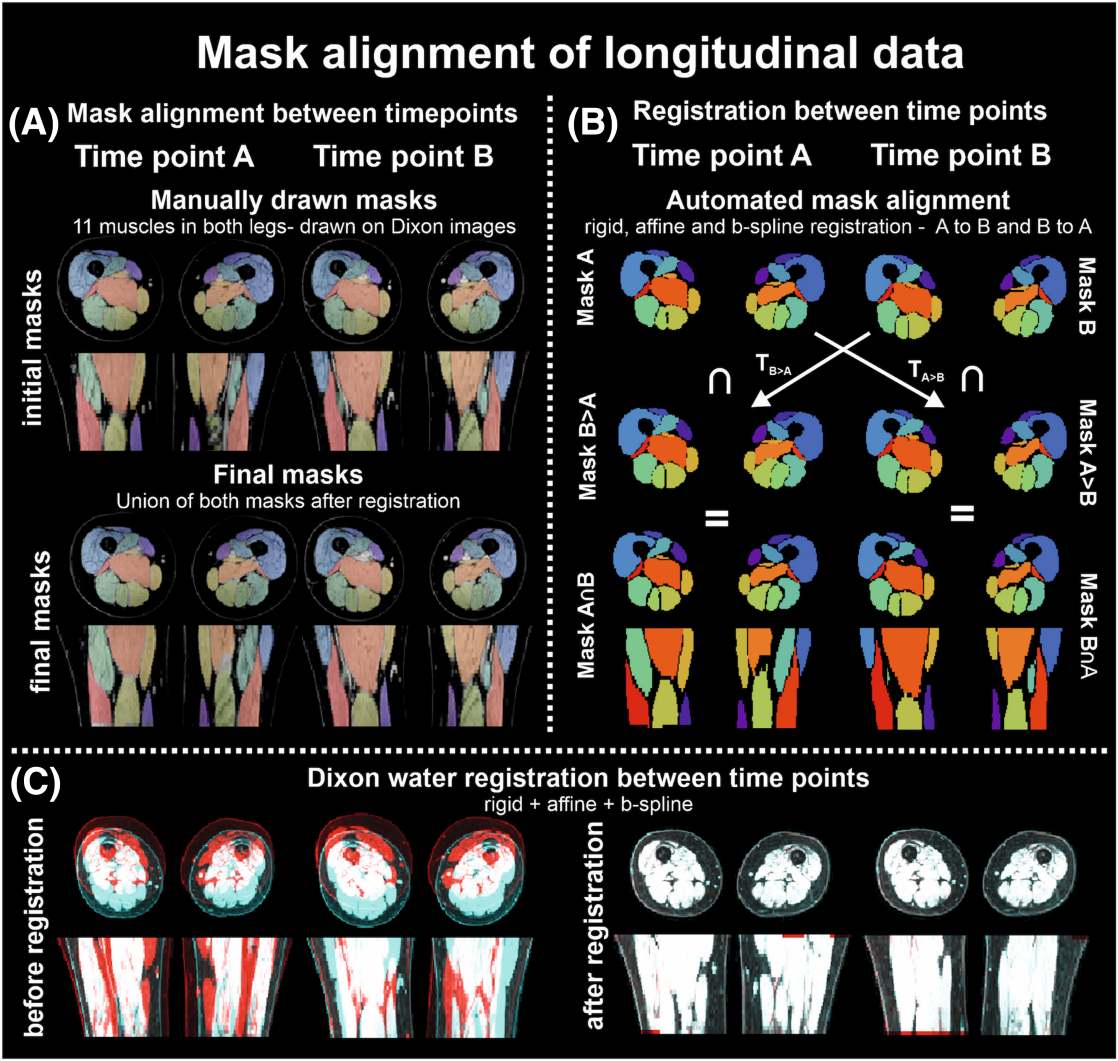
**Illustration of pipeline and steps of mask alignment of longitudinal data** The methodology of alignment of imaging stacks is illustrated by the steps involving conversion of masks; initial and final masks in panel A and automated steps in mask alignment in panel B. In panel C, the images of time‐point A and time‐point B are rendered red and blue, to illustrate the incongruency between datasets before (left) and after (right) rigid, affine and b‐spline registration. The non‐corresponding regions can be identified as they maintain their respective color

We aligned imaging stacks from baseline (Scan A) and follow‐up scans (Scan B) using multiple converting steps to ensure a match of the muscle segmentations at the two time‐points. Although care was taken to position and plan Scans A and B similarly, they are not identical (Figure 3). To select the corresponding anatomy between scans, non‐corresponding regions of the muscle segmentations were removed. This was done by registration of the Dixon water image of Scan A to Scan B using a combined rigid, affine and *b*‐spline registration. With the known transformation of Scan A to Scan B the manual segmentation was transformed from the image space of Scan A to the image space of Scan B. Next the union of both the transformed segmentations of Scan A and the native segmentation of Scan B was taken (Figure 3B). This step ensured the match of voxels, as the shape and position of the leg may vary between scans (Figure 3C). Also, the inverse of the transformation was applied to the segmentations of Scan B to move them to the image space of Scan A, and the union of the native segmentations of Scan A and transformed segmentations of Scan B was taken. This then led to a match between the two segmentations in the image space of Scan A and Scan B, to directly compare segmented muscles at the two time‐points at exactly the same level and location. Since all data were analyzed in the native space of that dataset, not all muscles contained sufficient voxels for analysis after registration. Therefore, the number of segmented muscles *N* reported could vary for each analysis.

### 2.6 Statistical analysis

Clinical scores and the fat fraction of muscle groups were compared between baseline and follow‐up using a paired *t*‐test. In the case of missing data, pairwise deletion followed. The change over time in qMRI outcomes was examined using a linear mixed effects model with two random intercepts for *subjects* and *muscle groups* according to an unstructured covariance matrix. Follow‐up time as a factor and baseline score were incorporated as fixed effects, with the addition of SNR for DTI parameters. We used the Wald statistic to determine whether a significant change over time occurred. A similar model was created for myometry of the three muscle groups. The threshold of significance was set at *p* < 0.05. Statistical analysis was performed using SPSS version 25 for Windows (SPSS, Chicago, IL).

#### 2.6.1 Data availability statement

The data that support the findings of this study are available from the corresponding author upon reasonable request.

## 3 RESULTS

We included 10 treatment‐naïve patients, ie none started with nusinersen or other SMA specific treatments during follow‐up. Patient characteristics are presented in Table 1. The mean follow‐up duration was 13.1 months, of which time between scans was at minimum 368 days and at maximum 442 days. The full MR protocol could be executed at baseline and follow‐up in all 10 selected patients, resulting in a complete dataset of 20 MR scans in total. All but one patient completed the repeated clinical measurements. Muscle ache after the study visit was the only reported adverse event, in one patient, and resolved spontaneously.

### 3.1 MR processing

After visual inspection of the data no datasets were excluded because of data quality or motion artifacts. SNR of the DTI data did not differ significantly between datasets (mean SNR 17.3 ± 6.8 at first scans, 17.7 ± 8.2 at second scans, mean difference −0.0 ± 4.7, *p* = 0.25).

### 3.2 Quantitative MR markers over time

**FIGURE 4:**
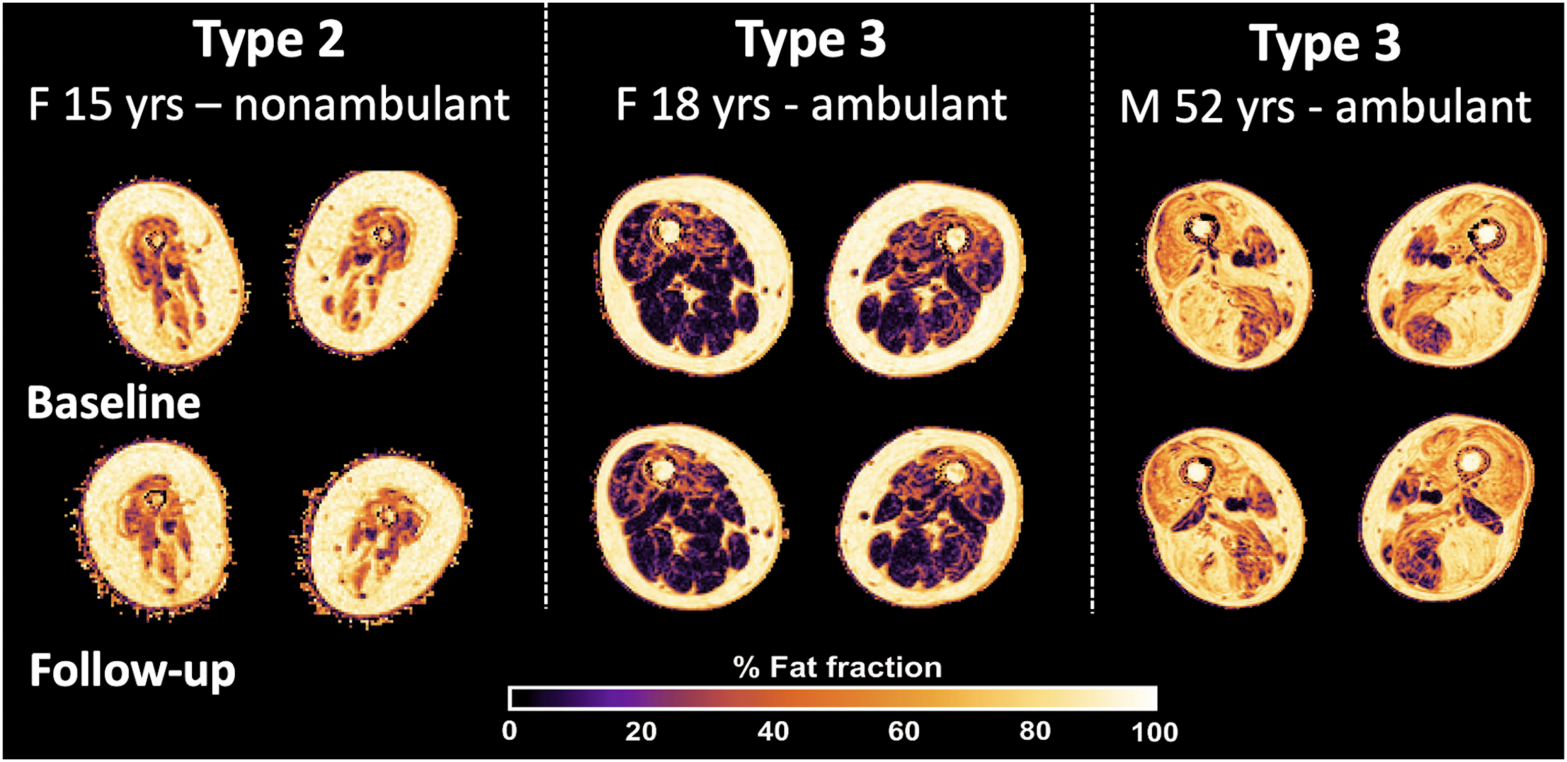
**Visual overview of fat infiltration at baseline and at follow‐up for type 2 and type 3** The color bar at the bottom indicates the gradient of fat fraction, ranging from lesser fat fraction in dark tones fading to lighter colors indicating higher fat fraction

**FIGURE 5:**
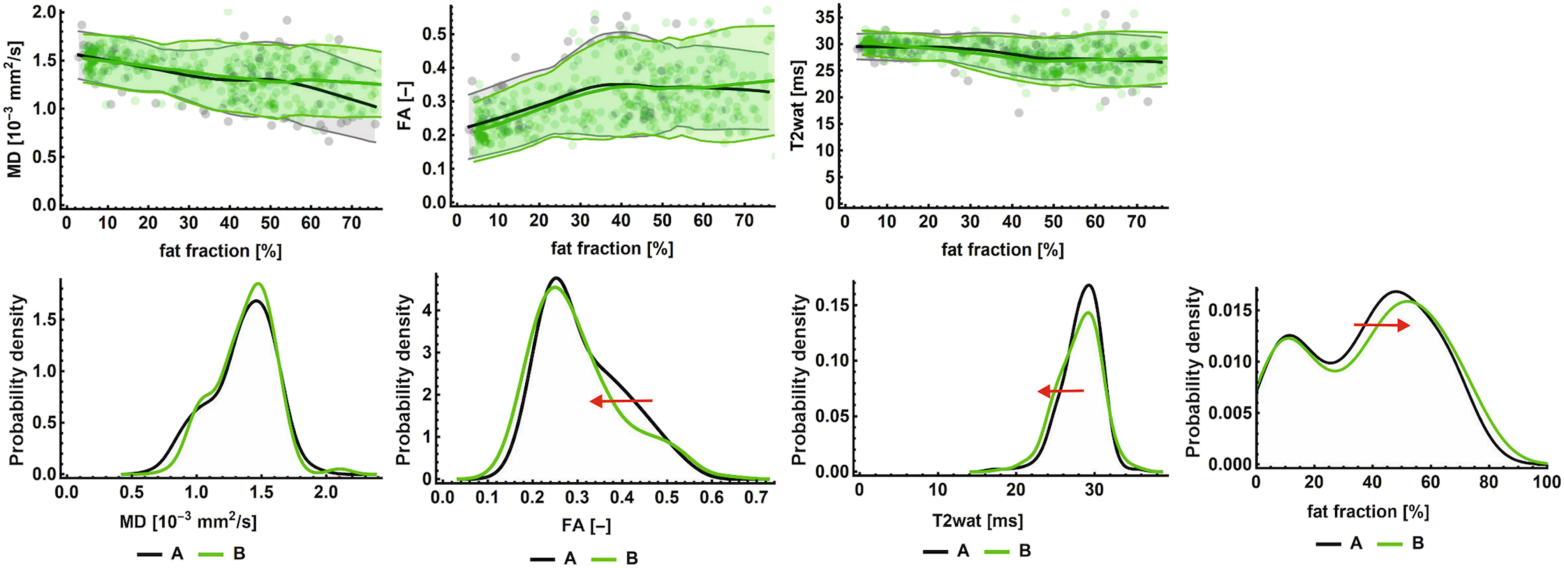
**Histogram and plots of qMRI parameters at both time‐points** In the upper row, MD, FA and T2 are plotted against fat fraction, with each of the individual datapoints as dots, reduced to an average line using local regression with 95%‐CI (shaded area). The bottom row represents the histograms of each of the qMRI parameters, the red arrow indicates significant changes and its direction. Timepoint A is indicated in grey, and time‐point B in green

Results from the linear mixed model of each of the qMRI parameters at Time‐points A and B are given in Table 3, visually presented for Type 2 and Type 3 (Figure 4) and plotted against fat fraction (Figure 5).

**TABLE 3:** qMRI parameters over time

qMRI parameter	Muscle *N*	Time‐point A mean (SE)	Time‐point B mean (SE)	Difference (SE)	95% CI	*P*	
*All muscles*						
Fat fraction (%)	402	38.22 (0.64)	39.50 (0.64)	1.28 (0.39)	0.51‐2.05	0.001
CSA (cm^2^)	401	6.21 (0.12)	5.95 (0.12)	−0.25	−0.61‐0.10	0.15
c‐CSA (cm^2^)	401	4.12 (0.06)	3.90 (0.06)	−0.23	−0.41‐−0.05	0.016
*T*_2_ (ms)	386	28.21 (0.16)	27.97 (0.16)	−0.24 (0.14)	−0.51‐0.02	0.074
MD (10^−3^ mm^2^/s)	360	1.35 (0.01)	1.37 (0.01)	0.02 (0.01)	−0.01‐0.04	0.121
FA	360	0.32 (0.01)	0.31 (0.01)	−0.01 (0.00)	−0.02‐0.00	0.007
*Muscles without m. adductor longus and m. biceps femoris (short head)*						
Fat fraction (%)	337	40.37 (0.73)	41.96 (0.73)	1.59 (0.42)	0.76‐2.4	<0.001
CSA (cm^2^)	336	6.49 (0.13)	6.20 (0.13)	−0.28 (0.18)	−0.67‐0.10	0.143
c‐CSA (cm^2^)	336	4.24 (0.07)	4.00 (0.07)	−0.25 (0.09)	−0.44‐−0.05	0.017
*T*_2_ (ms)	325	28.02 (0.17)	27.66 (0.17)	−0.36 (0.15)	−0.65‐0.06	0.018
MD (10^−3^ mm^2^/s)	302	1.34 (0.01)	1.36 (0.02)	0.02 (0.01)	−0.01‐0.05	0.114
FA	302	0.31 (0.01)	0.31 (0.01)	‐ 0.01 (0.00)	−0.02‐0.00	0.106
*Analysis of muscles, m. adductor longus and m. biceps femoris (short head) only*						
Fat fraction (%)	65	27.03 (0.86)	26.54 (0.89)	0.49 (1.02)	−2.55‐1.56	0.631
CSA (cm^2^)	65	4.76 (0.12)	4.65 (0.12)	−0.11 (0.12)	−0.36‐0.14	0.355
c‐CSA (cm^2^)	65	3.49 (0.10)	3.36 (0.10)	−0.13 (0.08)	−0.29‐0.03	0.110
*T*_2_ (ms)	61	29.23 (0.30)	29.61 (0.30)	0.38 (0.30)	−0.22‐0.99	0.211
MD (10^−3^ mm^2^/s)	58	1.43 (0.02)	1.44 (0.02)	−0.00 (0.02)	−0.04‐0.05	0.856
FA	58	0.33 (0.01)	0.29 (0.01)	−0.03 (0.01)	−0.06‐ −0.01	0.003

c‐CSA, contractile cross‐sectional area; CI, confidence interval; CSA, cross‐sectional area; *N*, number.

Results from the linear mixed model after correction for baseline. All data are represented by mean with standard errors, unless otherwise stated. The number of muscles that could be segmented is reported per qMRI parameter in the second column.

Mean fat fraction was 38.2% (SE 0.6) at baseline, and measured 39.5% (SE 0.6) at follow‐up. Combined analysis showed an increase in fat fraction of all muscles over time (slope +1.3%/time; 95% CI 0.51‐2.05) but only hamstrings showed a significant increase in fat fraction when analyzing muscle groups individually (from 37.1% to 38.7% (*p* = 0.04); adductors increased from 33.1 to 34.1% (+1.0%, *p* = 0.47) and quadriceps muscles from 44.0 to 44.5% (+0.5%, *p* = 0.40).

The histogram (Figure 5) of fat fraction showed a bimodal distribution, reflecting muscles with relatively high and low fat infiltrations. The m. adductor longus and the short head of m. biceps femoris are consistently less fat infiltrated than other muscles, with a fat fraction of 30% or less (see Table 4). When we excluded these two muscles from the mixed model analysis, the slope of fat fraction over time increased to +1.6% (95% CI 0.8‐2.4, *p* < 0.001). Change of fat fraction of the adductor longus and biceps femoris over time was −0.5% (95% CI −2.5‐1.5, *p* = 0.63). Removal of other muscles from the generalized linear model did not change the outcome. Subsequently, results from other qMRI parameters were analyzed with and without the incorporation of these two muscles.

**TABLE 4:** Fat fraction, CSA and c‐CSA of muscles at time‐point A and B

Muscle	Fat fraction (%)—A mean ± SD	CSA (cm^2^)—A total	c‐CSA (cm^2^)—A total	*n*—A	Fat fraction (%)—B mean ± SD	CSA (cm^2^)—B total	c‐CSA (cm^2^)—B total	*n*—B
Adductor longus	24.49 ± 14.39	75.87	53.33	17	24.87 ± 14.88	69.84	47.38	17
Adductor magnus	40.20 ± 19.34	203.15	145.42	17	42.97 ± 28.92	189.43	132,98	17
Biceps femoris (long)	39.05 ± 19.34	113.99	78.94	18	40.23 ± 20.77	111.53	76.27	18
Biceps femoris (short)	30.73 ± 25.47	78.01	59.29	16	28.19 ± 25.64	78.71	59.71	15
Gracilis	32.33 ± 20.05	63.72	44.20	19	33.89 ± 22.59	55.62	38.44	18
Rectus femoris	38.01 ± 20.37	58.84	40.32	17	37.65 ± 19.87	58.03	39.84	17
Sartorius	41.46 ± 18.77	45.61	27.68	16	41.92 ± 18.61	44.87	26.73	16
Semimembranosus	41.09 ± 19.17	118.58	76.87	20	42.69 ± 20.98	110.72	71.01	20
Semitendinosus	38.22 ± 17.42	79.72	49.22	20	41.25 ± 20.44	77.54	47.32	20
Vastus intermedius	37.51 ± 24.84	71.75	56.41	6	35.16 ± 23.74	65.72	51.97	6
Vastus lateralis	47.94 ± 23.09	225.27	127.24	16	49.31 ± 24.32	212.81	119.41	16
Vastus medialis	47.82 ± 21.42	109.58	66.93	20	49.20 ± 22.31	107.48	63.29	20
Total—all muscles	38.38 ± 21.34	1244.07	825.86	202	39.42 ± 22.60	1182.29	774.34	200

c‐CSA, contractile cross‐sectional area; CSA, cross‐sectional area; *n*, number of measurements per Time‐point A/B; SD, standard deviation. Mean values are descriptive, cross‐sectional means.

Although the histograms from Time‐points A and B mostly overlapped, there were significant changes in *T*_2_ and FA over time (Figure 5). *T*_2_ decreased from 28.2 ms (SE 0.2) to 28.0 ms (SE 0.2, *p* = 0.07), and further decreased after the exclusion of the m. adductor longus and m. biceps femoris (short head) (difference −0.4 ms, *p* = 0.02). FA decreased from 0.32 (SE 0.01) to 0.31 (SE 0.01, *p* < 0.01) when all muscles were analyzed simultaneously. However, when analyzed individually only the FA of the adductor longus and biceps femoris (short head) showed a significant decrease of 0.03 (*p* < 0.01). MD was not significantly different between time‐points, with or without the exclusion of the two aforementioned muscles. The outcome of these analyses did not change after correction for SNR, which is expected since there was no significant difference in SNR between timepoints.

### 3.3 Clinical assessments at baseline and follow‐up

Clinical assessments are summarized in Table 5. Hamstrings generated the highest mean muscle force, followed by the adductors and lastly the quadriceps, as measured by HHD. HFMSE, MRC scores and HHD were not significantly different between baseline and follow‐up.

**TABLE 5:** Clinical measurements at baseline and follow‐up

Clinical measurements	Baseline	Follow‐up	*p*
HFMSE score			
mean (SD) [score range 0‐66]	27.9 (27.7)	25.1 (27.6)*	0.364
MRC sum score			
mean (SD) [score range 44‐210]	142.7 (41.6)	141.6 (41.8)*	0.257
MRC sum score thigh			
mean (SD) [score range 6‐30]	16.3 (5.7)	16.5 (5.7)	0.169
HHD of muscle group (N)			
mean [95% CI]			
Adductors	39.4 [8.0‐70.9]	45.6 [14.2‐77.1]	0.221
Hamstrings	47.1 [5.0‐89.1]	59.1 [17.1‐101.1]	0.058
Quadriceps	16.0 [−7.9‐39.8]	20.2 [−3.6‐44.1]	0.226

* *n* = 9; CI, confidence interval; N, Newton; SD, standard deviation.

Clinical characteristics are reported for patients with SMA Type 2 and Type 3. Clinical measurements are reported as means with standard deviation, or the 95% CI where we used a mixed model. *p*‐values of paired *t*‐testing and of the mixed model of clinical measurements are reported.

## 4 DISCUSSION

We here show that qMRI parameters obtained from thigh muscles of patients with SMA Types 2 and 3 change significantly in the course of a year, whereas strength and motor function remain unchanged. Previous studies consistently showed that clinical assessments lack sensitivity to detect changes in follow‐up periods shorter than 2‐5 years.^8^, ^11^, ^12^, ^30^, ^31^, ^32^ This study, therefore, demonstrates that qMRI can detect subclinical disease progression and is a promising biomarker.

Comparison of longitudinal imaging datasets is challenging and may impede clinical application. When comparing structures between imaging stacks or analyzing whole muscle volume, two components are of importance; accuracy of segmentation and match of anatomical locations. Slice‐by‐slice manual segmentation has already demonstrated good‐to‐excellent reliability.^33^ For the latter, we describe in this study a method to overcome the misalignment that is inherent to comparing scans from two time‐points. We chose to segment and analyze all muscles separately and only use those regions that were present and consistently segmented in consecutive scans. In this way, only muscle tissue that was present in both examinations at the same location was used for analysis. With this approach a whole muscle can be compared individually, whilst retaining its multitude of information and without subsampling or deformation of the parameter maps. Our methodology thus allowed us to include all 25 slices of the imaging stack, spanning 15 cm, which benefitted accuracy and statistical power. In comparison, a previous longitudinal study in SMA that employed the Dixon sequence and compared three slices of the imaging stack failed to detect significant changes (+2.3% after a year).^13^

The pace of fatty degeneration in SMA is slow and comparable to that in other neuromuscular diseases, which are generally characterized by a yearly progression rate of less than 5%.^15^, ^21^, ^34^, ^35^ Other longitudinal studies in muscular dystrophies show that only a few functional tests were able to detect changes in the timespan over which fat infiltration progressed on MRI.^15^, ^21^, ^36^, ^37^, ^38^ SMA is furthermore characterized by a higher overall fat infiltration, although there are few longitudinal studies in treatment‐naïve adult patients with other neuromuscular diseases for comparison.^15^, ^20^, ^21^, ^34^

The increase in fat fraction seems to occur at the cost of contractile muscle tissue, as illustrated by the significant decrease in contractile cross‐sectional area over the course of a year. In line with previous clinical observations,^8^, ^9^, ^39^ qMRI data show clear differences between muscles. The pattern of relatively vulnerable and spared muscles that is characteristic of SMA is reflected by the bimodal distribution of fat fraction. Two separate peaks indicate the difference between low and high levels of fatty infiltration. This difference is clearly muscle specific but probably not static. Data from a cross‐sectional study in SMA indicate that the adductor longus and biceps femoris muscles eventually show fatty replacement exceeding 30% of muscle volume up to end‐stage full fatty replacement.^23^ The lack of a clear spectrum may suggest that fatty infiltration is not a continuous process but can rather quickly convert at some tipping point towards high fatty content. This hypothesis is also supported by the finding that fat infiltration occurs at a slow yearly speed in muscles with less than 30% of fat infiltration as compared with a rapid yearly increase in muscles with fat infiltration greater than 30%.

Also, we noticed a very small (−0.2 ms) but significant decrease in *T*_2_ over time. We think the decrease is related to the bias of a simultaneous increase of fat fraction, as increasing fat replacement results in lowering of *T*_2_, as we observed in our previous study.^23^, ^28^ Although *T*_2_ mapping is often used as a meaningful outcome measure in other neuromuscular diseases, its application as a biomarker for SMA therefore seems irrelevant, although its value in monitoring treatment effect remains to be determined.

The DTI measures MD and FA did not show significant changes over time in moderate to severely affected muscles. We observed a small significant change in FA only in low fat‐infiltration muscles, ie the adductor longus and biceps femoris (short head), that persisted after correction for SNR.^40^ The decrease in FA in probably does not represent increased permeability of cell membranes because MD values did not change accordingly. Possible alternative explanations for the observed FA decrease in low fat‐infiltration muscles are increased strain while other muscles deteriorate, resulting in swelling. FA decrease may also reflect a distinct moment in (early) muscle pathology. Whether DTI changes are preceding fat infiltration remains inconclusive and has to be demonstrated by additional measurements in an early phase of muscle pathology in young children. Because of the small standard deviation of MD and FA (0.1 and 0.2 respectively) at both time‐points, we hypothesize that DTI can still be a sensitive measure for evaluating treatment effects despite not qualifying as a biomarker for disease progression in untreated patients.

In the cross‐sectional cohort, which was a inhomogeneous group that included patients from 7 to 73 years, the overall change of FA was 0.2 over the span of 60 years.^23^ Now, we observe that FA, similar to MD, is not sensitive as a measure for change within a year. Nonetheless, previous work has also shown that FA in affected muscle is different from healthy muscle, and that what is considered healthy muscle in SMA has comparable FA values to muscle of controls.^23^ This broad range of FA and the possibility for normalization of FA values holds potential when monitoring treatment response.

All clinical measurements were done by one evaluator. Previous studies have shown that the intra‐rater reliability of these measurements in SMA are 0.959 for HFMSE^41^ and more than 0.91 for HHD.^42^ Additionally, research in DMD showed that MRC reliability within a study improved when consecutive evaluations were done by the same evaluator.^43^ Clinical measures appear insensitive to minor changes, as reflected by the stable MRC score, or can show day‐to‐day variation, as illustrated by the HHD results. We thus propose MRI as a more objective method for monitoring patients. Good correlation of Dixon‐FF and moderate correlation of DTI parameters with clinical measures has been established in a cross‐sectional study in SMA.^23^

The small sample size is a clear limitation to this study. However, SMA is rare and the fact that patients are often severely impaired complicates MRI studies. Muscle MRI of lower extremities in SMA therefore seems limited to young and adolescent patients with Type 2 and young to adult patients with Type 3. qMRI of the upper extremities could represent a future alternative, after the solution of some logistic and technical issues including positioning of the patient (especially those with contractures) and the fact that arms cannot be imaged simultaneously, resulting in prolonged scanning time. This study on natural history of disease progression excluded patients who started treatment; they are still undergoing follow‐up measurements. There will be fewer future opportunities to gather reference data from treatment‐naïve patients, since treatment is becoming widely available.

To conclude, longitudinal imaging data show slow disease progression in skeletal muscles of the thigh of (young‐) adult patients with SMA despite stable strength and motor function scores. As multiple disease‐modifying therapies have become available since the start of this study, this dataset, on treatment‐naïve patients, provides insight into the natural history of SMA. This pilot study demonstrates the potential of qMRI as a biomarker for disease activity and monitoring of therapy response.

## CONFLICTS OF INTEREST

WLP is a member of the scientific advisory board of SMA Europa and has served as an ad hoc member of the scientific advisory boards of Biogen and Avexis and as a member of a data monitoring committee for Novartis. WLP receives grants from Prinses Beatrix Spierfonds and stichting Spieren voor Spieren. The other authors report no conflicts of interest.
